# Correction to: Differential Effects of Prenylation and *S*-Acylation on Type I and II ROPS Membrane Interaction and Function

**DOI:** 10.1093/plphys/kiaf089

**Published:** 2025-09-24

This is a **correction** to:

Nadav Sorek, Orit Gutman, Einat Bar, Mohamad Abu-Abied, Xuehui Feng, Mark P. Running, Efraim Lewinsohn, Naomi Ori, Einat Sadot, Yoav I. Henis, Shaul Yalovsky, Differential Effects of Prenylation and *S*-Acylation on Type I and II ROPS Membrane Interaction and Function, *Plant Physiology*, Volume 155, Issue 2, February 2011, Pages 706–720, https://doi.org/10.1104/pp.110.166850

This correction addresses the issues raised on Pubpeer (https://pubpeer.com/publications/09890676701890565C32F05A7F3E99) concerning our publication titled “Differential Effects of Prenylation and S-Acylation on Type I and II ROPs Membrane Interaction and Function.” Plant Physiology, 155: 706-720. doi: 10.1104/pp.110.166850.

The issues raised pertain to Figures 3A, 4, and 5A. A concern on Figure 2A has been addressed in 2015 (https://doi.org/10.1016/j.cub.2015.10.029).

Dr. Nadav Sorek, the first author of the paper, prepared the figures and acknowledged that he is responsible for all of the errors. Following careful examination of the errors and comparison with the raw data, we concluded that the conclusions in the paper are still valid.

**Figure 3A.** The figure depicts purified fractions of PGGT-I (protein geranyl geranyl transferase-I), PFT (protein farnesyl transferase), and ROP6 that were employed in *in vitro* prenylation reactions. The issue raised concerns the removal of background at the top of the PFT (Protein Farnesyl Transferase) lane.

**Correction.** The elimination of the background at the top of the PFT lane was a misguided attempt to remove the shaded area at the top of the gel, likely caused by adhesive tape, as well as to align the tilted lane. This action was unequivocally incorrect and should not have occurred, as there was no justification for it. We sincerely apologize for this mishandling of the data. To address these concerns, we are presenting the original image of the Coomassie blue-stained gels.

**Figure 3A.**
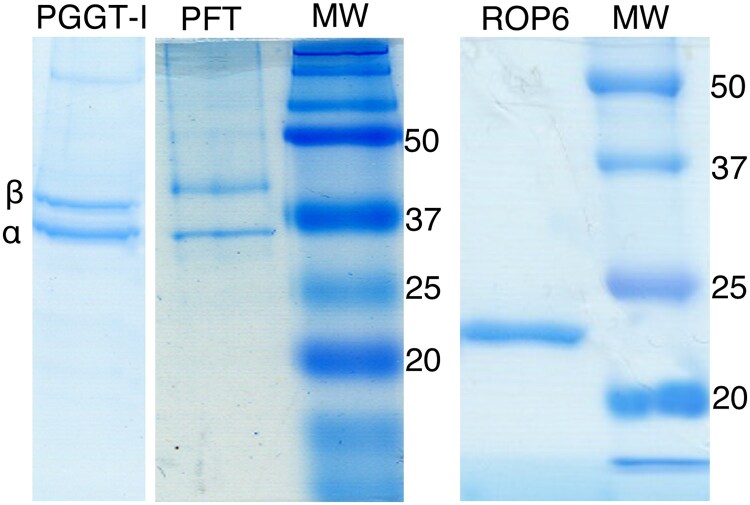
Coomassie blue stained SDS-PAGE gels showing purified recombinant PGGT-I, PFT, and ROP6. MW denotes size markers. α and denote the common α and the unique β subunits of PGGT-I and PFT, respectively.

**Figure 5A.** This issue addresses duplication of the FRAP curve on the top right (63X 6WT, WT) to the bottom right panel (63X 6CA, ggb). In addition, we noticed that the units of the X axis in the original figure are incorrect: they should be seconds and not minutes as incorrectly labeled in the original figure

**Correction.** This figure displays representative FRAP (Fluorescence Recovery After Photobleaching) curves, in each case showing one curve out of 35 made and averaged together in panels B and C.

It appears that during the preparation of the figure, an effort was made to select curves that appeared most similar, although there was no specific reason to do so as the Y scale is in relative arbitrary units. During the figure's composition, the curve shown in the top right panel was also placed in the bottom right panel. We should note that the FRAP curves in the current set of experiments have a constant background fluorescence level (autofluorescence of the glass surface) of about 80. The curves are available either with or without this background, and we usually show the background-subtracted curves. It appears that in the case of the curve in the top right panel, the curve without this subtraction was taken, which caused the curve on the right bottom panel to be shifted by about 80 units downward on the Y axis. We apologize for not noticing this in the original version. Finally, the scale on the time axis of this figure was labeled erroneously as minutes, while it should be seconds, as is evident from the τd values.

To rectify this, we have now replaced all sample FRAP curves with other, unrelated sample curves from the same set of measurements, which are devoid of these errors.

**Figure 5A.**
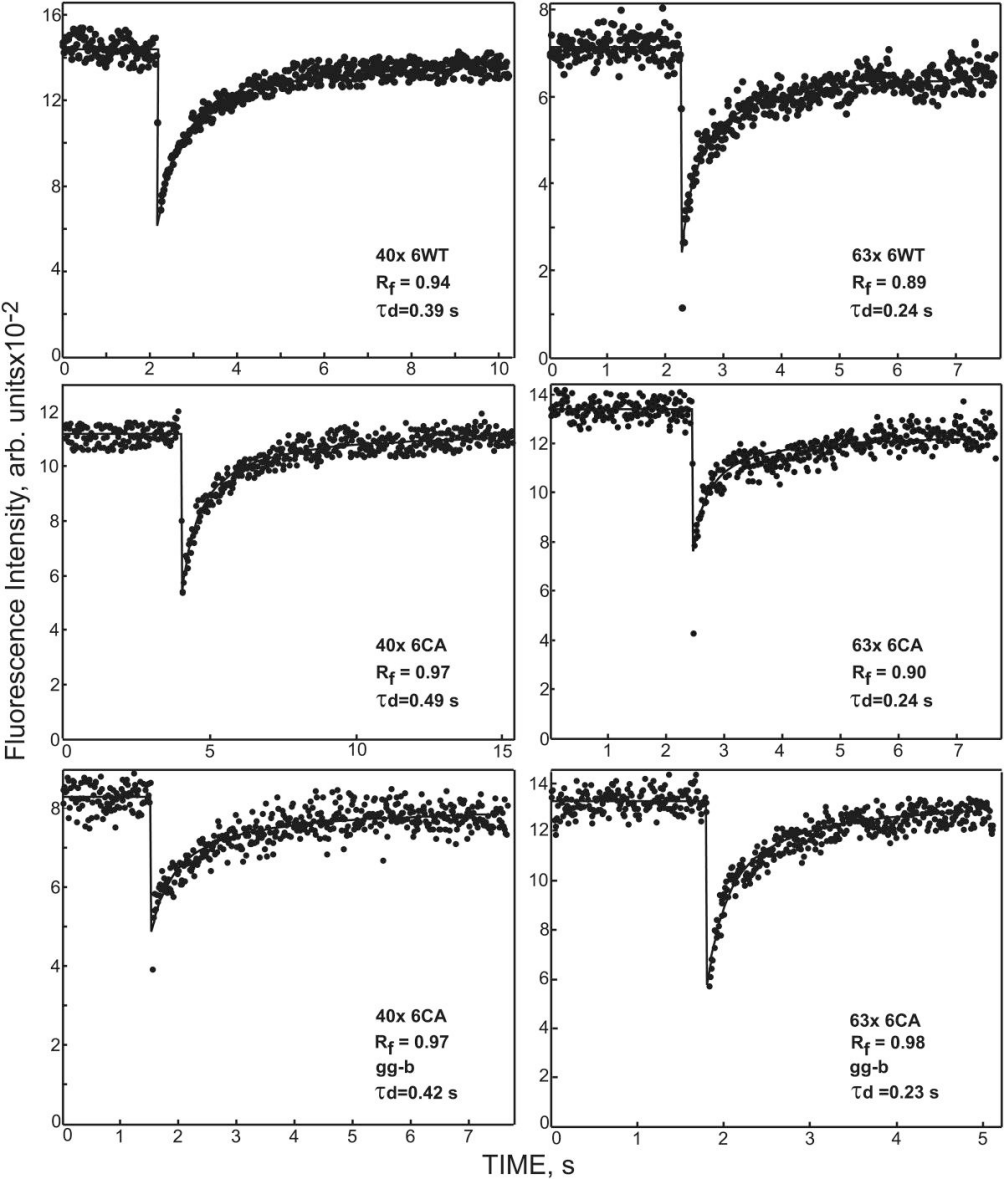
Typical FRAP curves obtained with either 40X or 63X objectives for His_6_-GFP-ROP6 (6WT), His_6_-GFP-rop6CA (6CA) in wild type or in pggt-Ib (gg-b) leaf epidermis pavement cells. The solid lines show the best fit of a nonlinear regression analysis to the lateral diffusion equation. The characteristic diffusion time (τd) and mobile fraction (R_f_) values derived for each specific curve are shown.

**Figure 4.** The issue raised here is that some of the GC-MS spectrometry spikes might be more similar than would be expected by chance.

**Response.** This figure presents GC-MS (gas chromatography-mass spectrometry) peaks, and it was suggested that some of the peaks appear very similar. We would like to clarify that this similarity is due to the narrowness of the peaks, and a close look at them shows that they are not, in fact, identical.

**Figure 2A.** There was a concern that Figure 2A is identical to a figure in paper that we published in Current Biology (Sorek et al. (2010) Current Biology, doi: 10.1016/j.cub.2010.03.057).

**Correction.** Already in 2015 we addressed this problem and submitted a revised version of Figure 3 to Current Biology. The revised Fig. 3C (CA) is a new image different from the image presented in Plant Phys Fig. 2A. Current Biology published a revised version of our paper with the correct Figure 3. (Sorek et al (2015) Current Biology, doi: 10.1016/j.cub.2015.10.029).

In summary, we extend our sincerest apologies for these inaccuracies in the preparation of the data for publication. This Correction aims to clarify the concerns raised regarding our paper, ensuring that its conclusions remain reliable and valid.

These details have been corrected only in this **correction notice** to preserve the published version of record.

9 December 2025 This correction notice has been amended to include a link to the comments raised on PubPeer regarding the original article, https://pubpeer.com/publications/09890676701890565C32F05A7F3E99, as well as a link to the 2015 article cited, https://doi.org/10.1016/j.cub.2015.10.029.

Additionally, the caption for Figure 5A was mistakenly duplicated in the originally published correction notice—appearing both as the figure caption and again in the main text. The figure caption in the main text has now been removed.

